# LMWF5A suppresses cytokine release by modulating select inflammatory transcription factor activity in stimulated PBMC

**DOI:** 10.1186/s12967-020-02626-z

**Published:** 2020-11-30

**Authors:** Gregory Thomas, Elizabeth Frederick, Lisa Thompson, Raphael Bar-Or, Yetti Mulugeta, Melissa Hausburg, Michael Roshon, Charles Mains, David Bar-Or

**Affiliations:** 1 Ampio Pharmaceuticals Inc, 373 Inverness Parkway Suite 200, Englewood, CO 80122 USA; 2 Trauma Research Department, Swedish Medical Center, 501 E. Hampden Ave. Rm 4-454, Englewood, CO 80113 USA; 3 Trauma Research Department, St. Anthony Hospital, 11600 W 2nd Pl, Lakewood, CO 80228 USA; 4 Trauma Research Department, Penrose Hospital, 2222 N Nevada Ave, Colorado Springs, CO 80907 USA; 5 Centura Health Systems, 9100 E. Mineral Cir, Centennial, CO 80112 USA; 6 Department of Molecular Biology, Rocky Vista University, 8401 S Chambers Rd, Parker, CO 80134 USA

**Keywords:** LMWF5A, PPARγ, AhR, NF-κb, STAT, Transcription, Cytokines, Chemokines

## Abstract

**Background:**

Dysregulation of transcription and cytokine expression has been implicated in the pathogenesis of a variety inflammatory diseases. The resulting imbalance between inflammatory and resolving transcriptional programs can cause an overabundance of pro-inflammatory, classically activated macrophage type 1 (M1) and/or helper T cell type 1 (Th1) products, such as IFNγ, TNFα, IL1-β, and IL12, that prevent immune switching to resolution and healing. The low molecular weight fraction of human serum albumin (LMWF5A) is a novel biologic drug that is currently under clinical investigation for the treatment of osteoarthritis and the hyper-inflammatory response associated with COVID-19. This study aims to elucidate transcriptional mechanisms of action involved with the ability of LMWF5A to reduce pro-inflammatory cytokine release.

**Methods:**

ELISA arrays were used to identify cytokines and chemokines influenced by LMWF5A treatment of LPS-stimulated peripheral blood mononuclear cells (PBMC). The resulting profiles were analyzed by gene enrichment to gain mechanistic insight into the biologic processes and transcription factors (TFs) underlying the identified differentially expressed cytokines. DNA-binding ELISAs, luciferase reporter assays, and TNFα or IL-1β relative potency were then employed to confirm the involvement of enriched pathways and TFs.

**Results:**

LMWF5A was found to significantly inhibit a distinct set of pro-inflammatory cytokines (TNFα, IL-1β, IL-12, CXCL9, CXCL10, and CXCL11) associated with pro-inflammatory M1/Th1 immune profiles. Gene enrichment analysis also suggests these cytokines are, in part, regulated by NF-κB and STAT transcription factors. Data from DNA-binding and reporter assays support this with LMWF5A inhibition of STAT1α DNA-binding activity as well as a reduction in overall NF-κB-driven luciferase expression. Experiments using antagonists specific for the immunomodulatory and NF-κB/STAT-repressing transcription factors, peroxisome proliferator-activated receptor (PPAR)γ and aryl hydrocarbon receptor (AhR), indicate these pathways are involved in the LMWF5A mechanisms of action by reducing LMWF5A drug potency as measured by TNFα and IL-1β release.

**Conclusion:**

In this report, we provide evidence that LMWF5A reduces pro-inflammatory cytokine release by activating the immunoregulatory transcription factors PPARγ and AhR. In addition, our data indicate that LMWF5A suppresses NF-κB and STAT1α pro-inflammatory pathways. This suggests that LMWF5A acts through these mechanisms to decrease pro-inflammatory transcription factor activity and subsequent inflammatory cytokine production.

## Background

Dysregulation of transcription and the expression of inflammatory proteins has been implicated in the pathogenesis of a variety of chronic diseases including arthritis, atherosclerosis, diabetes, pulmonary fibrosis, kidney disease, and inflammatory bowel disease [1]. Although these conditions exhibit a range of clinical symptoms, they all involve an overactivation of the immune response and/or an inability of the immune response to progress towards resolution and healing. Under normal circumstances, the immune system transitions through sequentially triggered sets or profiles of functionally related genes, known as expression programs, that drive the cellular activity and cell-type identity of immune cells in a return to homeostasis [2]. Early damage or infection signals induce the genetic orientation of classically activated type 1 macrophages (M1) and CD4+ helper type 1 T-cell (Th1) subsets, which produce pro-inflammatory cytokines and chemokines such as IFNγ, TNFα, IL1-β, and IL12 [3]. Once the insult has been eliminated, biologic feedback systems then promote the active conversion of immune cells to alternatively activated type 2 macrophages (M2) or CD4+ helper type 2 (Th2)/regulatory (Treg) T-cell subsets, transcriptionally programmed to produce anti-inflammatory mediators that dampen the inflammatory response and trigger a switch into resolution and healing phases [2, 3]. However, chronic inflammation can manifest under conditions where pro-inflammatory programs sustain the production of cytokine and/or resolving programs fail to eventuate [4].

Central to this level of regulation is the activity of transcription factors (TFs). Biochemical cascades, ushered by specific transmembrane and intracellular receptor-ligand interactions, evoke the transcription of target genes through the binding of TFs to cognate sequences located in DNA promoter regions [5]. Ultimately, cellular gene expression programs result from the complex interplay between multiple transcription factors and cell-specific regulatory machinery acting together at promoter elements in response to the microenvironment signals received [6]. This complexity enables the immune system to pivot and direct gene expression programs that are specifically crafted for each challenge or phase of the inflammatory response. Moreover, a large body of evidence demonstrates that DNA-binding motifs for TFs such as activator protein 1 (AP-1), nuclear factor kappa-light-chain-enhancer of activated B cells (NF-κB), and signal transducer and activator of transcription proteins (STAT) are over-represented in the promoters of pro-inflammatory genes, suggesting that inflammatory signaling is intimately linked to these regulatory proteins [6]. In support of this, exaggerated and protracted NF-κB and/or STAT signaling has been linked to chronic inflammation and many of the diseases listed above [7, 8]. It is also now well established that these TFs contribute to the production of pro-inflammatory M1/Th1 gene signatures and if left unchecked, can lead to excessive immune activation and tissue damage [9, 10]. Treatments that modulate or interrupt pro-inflammatory TFs may shift the overall response towards homeostasis and provide medical benefits to patients suffering from both acute and chronic inflammatory diseases.

To orchestrate the switching of inflammation to resolution programs and promote healing, transcriptional machinery has evolved that functions to suppress inflammatory signaling pathways [2]. Two such metabolic-associated TFs are peroxisome proliferator-activated receptor (PPAR)γ and aryl hydrocarbon receptor (AhR) [11]. These ligand-activated TFs have been shown to aid in coordinating the differentiation of anti-inflammatory and regulatory immune cell phenotypes while, at the same time, repressing the expression of pro-inflammatory cytokines from M1 macrophages [11, 12]. Pharmacologically activating these proteins has proven successful at reducing inflammation by both reducing the DNA-binding potential of pro-inflammatory TFs as well as modulating the activity of repressor and/or cofactor molecules [13–15]. More importantly, clinical application and repositioning of PPARγ and AhR agonists has shown promise in the treatment of chronic inflammation and cancer [16, 17].

The low molecular weight fraction of human serum albumin (LMWF5A) is a novel anti-inflammatory biologic drug that has demonstrated clinical efficacy with reduced pain and improved function in osteoarthritis of the knee, a chronic inflammatory condition, across multiple randomized, vehicle-controlled, double-blinded human trials [18–20]. The potential benefits of LMWF5A as an immunomodulatory therapeutic are also denoted in an observed delay in the need for total knee replacement for severe osteoarthritis trial participants in a 3-year follow-up study [21]. Based on these observations, LMWF5A may serve as an immunomodulatory agent for the treatment of a variety of inflammatory diseases. In fact, clinical studies have been launched to investigate the use of LMWF5A to treat the systemic inflammatory response syndrome and respiratory distress associated with COVID-19.

Previous in vitro investigations suggest that the clinical effects of LMWF5A may result from the transcriptional modulation of inflammatory mediators. For example, LMWF5A treatment of lipopolysaccharide (LPS)-stimulated peripheral blood mononuclear cells (PBMC) reduces TNFα transcription and release, concomitant with increased transcription and expression of COX2, as compared to saline-treated controls [22, 23]. In addition, phorbol 12-myristate 13-acetate (PMA)-differentiated THP-1 macrophages exhibit cytokine expression profiles and transcriptional patterns reflective of a switch from M1 to M2 polarization status when stimulated with LPS in the presence of LMWF5A [24]. Interestingly, LMWF5A also demonstrates an ability to transcriptionally modulate other cell functions; for example, chondrogenic differentiation [25, 26] and the homing/migratory potential of bone marrow derived mesenchymal stem cells [25]. Thus, we hypothesized that the mechanisms of action of LMWF5A involve the activation of regulatory transcription factors critical to both immune cell activation and differentiation.

To test this hypothesis, cytokine arrays and bioinformatic analysis were applied to the LPS-stimulated PBMC model utilized in our preceding studies, with the aim of identifying cellular and molecular mechanisms responsible for the observed ability of LMWF5A to influence transcription and protein expression. Identified in silico pathways were examined with pathway-specific pharmacologic antagonists and transcription factor/DNA-binding assays to elucidate mechanisms of action involved with the ability of LMWF5A to inhibit key pro-inflammatory cytokines. In this report, we provide evidence that LMWF5A activates the immunoregulatory transcription factors PPARγ and AhR as well as suppresses the classical NF-κB and STAT1α pro-inflammatory signaling pathways. These data provide biologic rationale for the anti-inflammatory properties of LMWF5A and suggests LMWF5A could be a therapeutic treatment for a variety of inflammatory conditions.

## Methods

### Materials

Cell culture reagents were purchased from Gibco, ThermoFisher Scientific (Waltham, MA), and all other reagents were obtained from MilliporeSigma (St. Louis, MO) unless otherwise stated. 5% human serum albumin (Octapharma, Hoboken, NJ) was used to produce LMWF5A. 0.9% (w/v) sodium chloride was obtained from KD Medical (Columbia, MD). 100 mM stock solutions of GW9662 and CH223191 were prepared in DMSO and stored at −80 °C prior to use. ELISAs for TNFα (catalog# DTA00D) and IL-1β (catalog# SLB50) were purchased from ThermoScientific and R&D systems (Minneapolis, MN), respectively.

### PBMC culture and experimental treatment

Commercially available frozen human peripheral blood mononuclear cells (Zen-Bio, Research Triangle Park, NC) from a single, strong LMWF5A response donor were used for the ex vivo experiments described in this report. Cell vials stored in liquid nitrogen were thawed using a Thawstar Automated Cell Thawing System (BioLife Solutions, Bothell, WA) and then transferred dropwise to RPMI 1640 medium containing 10% human AB serum, 1% penicillin–streptomycin (Pen/Strep), and 2 U/mL RNase-free DNase (ThermoScientific). The resulting cell suspensions were centrifuged (at 1000 rpm for 10 min) and the medium was replaced with RPMI 1640 supplemented with 20% fetal bovine serum, 2% Pen/Strep, 1% sodium bicarbonate 7.5% solution, 1% 100 mM sodium pyruvate, 1% 100X MEM non-essential amino acid solution, and 1% 200 mM L-glutamine. For experimental treatments, cell suspensions were adjusted to 2 × 10^6^ cells/mL using the described culture medium and then mixed with equal volumes of sterile 0.9% sodium chloride or LMWF5A and incubated at 37 °C and 5% CO_2_ for one hour. Cells were stimulated with O55:B5 lipopolysaccharide (final concentration of 100 ng/ml; catalog# L6529) and incubated for an additional 24 h before subsequent analysis.

### Cytokine arrays

To evaluate large-scale release of cytokines and chemokines, saline- or LMWF5A-treated, LPS-stimulated PBMC were cultured as described above in triplicate in 0.8-mL final reaction volumes for 24 h and then centrifuged (1000 rpm, 10 min). The resulting conditioned mediums were combined, and cytokine release was analyzed using Human Cytokine ELISA Plate Array IV (Signosis, Santa Clara, CA; catalog# EA-4015) following the manufacturer’s instructions, with optical density (OD) measured at 450 nm using an ELx808 Absorbance Microplate Reader (Biotek Instruments, Winooski, VT). Fold change in blank-subtracted OD measurements of LMWF5A-treated, LPS-stimulated cultures versus saline-treated, LPS-stimulated groups were then calculated for three independent experiments. Enrichment analysis of differentially abundant cytokines was performed using the web-based applications Enricher (https://amp.pharm.mssm.edu/Enrichr/) and Chea3 (https://amp.pharm.mssm.edu/chea3/).

### Transcription factor DNA-binding ELISAs

To establish the activity of select transcription factors, saline- or LMWF5A-treated, unstimulated or LPS-stimulated PBMC were cultured as described above in a final reaction volume of 25 mL for 24 h and then centrifuged at 1000 rpm for 10 min. Nuclear protein was extracted from the cell pellets using a nuclear extraction kit (Active Motif, Carlsbad, CA), and the captured protein concentration was determined using Pierce detergent-compatible Bradford reagent (ThermoScientific). DNA-binding ELISAs (Active Motif) were then performed for NF-κB subunits (catalog# 43,296), AP-1 subunits (catalog# 44,296), and STAT (catalog# 42,296) using 2 µg, 5 µg, and 5 µg total protein per reaction, respectively, following the manufacturer’s recommendations.

### Relative potency bioassays

To determine the potency of LMWF5A test samples, LPS-stimulated PBMC were cultured as described above in quadruplicate in 110 µL final reaction volumes, blocked to protect against location bias, in 96-well tissue culture plates with five, 1.4-fold serial dilutions of LMWF5A (prepared in saline). After 24 h, the plates were centrifuged at 1,000 rpm for 10 min, and TNFα and IL-1β release was measured by ELISA following the manufacturer’s instructions. Saline-treated, LPS-stimulated controls were also included for calculating % inhibition of cytokine release. PLA 3.0 bioassay software (Stegmann Systems, Rodgau, Germany) was used to calculate relative potency as compared to similarly prepared LMWF5A reference material tested on each plate.

### HEK293 luciferase reporter assay

HEK-Dual TNFα cells (Invivogen, San Diego, CA; catalog #hkd-tnfa) were used to assess the ability of LMWF5A to inhibit NF-κB. These cells contain a reporter construct that encodes a secreted luciferase gene (Lucia) under the control of a NF-κB inducible promoter. 130 µL LMWF5A was added in triplicate to a 96-well plate. 70 µL HEK-Dual TNFα cells in DMEM (Corning, Manassas, VA) supplemented with 10% heat-inactivated FBS, 10 U/mL pencillin-100 µg/mL streptomycin, 100 µg/ml normocin (Invivogen), and 100 µg/ml zeocin (Invivogen) were then added for a final concentration of 50,000 cells/well, and the cells were incubated at 37 °C and 5% CO_2_ for 24 h. To induce luciferase expression, the cells were stimulated with 2 ng/mL TNFα (Invivogen) for an additional 24 h at 37 °C and 5% CO_2_. The luciferase activity was quantitated by combining 20 µL each cell supernatant and 100 µL QUANTI-Luc reagent (Invivogen) in a new, opaque 96-well plate and immediately measuring luminescence on a SpectraMax M5e and Flexstation 3 System (Molecular Devices, San Jose, CA).

### Data and statistical analysis

Statistical analysis was performed using the Real Statistics Resource Pack Excel Add-in (http://www.real-statistics.com/) unless otherwise stated. For cytokine arrays, one-tailed, one-sample t-tests (hypothetical value = 0; α = 0.05) were used to establish meaningful OD measurements above medium-control blanks and two-tailed, one-sample t-tests were used to test for the significance of combined fold changes (hypothetical value = 1; α = 0.05). For DNA-binding ELISAs, two-tailed, two-sample unequal variance student tests were used for the representative ELISAs in Microsoft Excel (Microsoft Corporation, Redmond, WA) and Wilcoxon signed-ranked tests were used for non-parametric analysis of combined fold changes (hypothetical value = 1; α = 0.05). For potency assays, relative potency was calculated using parallel-line analysis with ANOVA with pure separation and similarity of sample responses was established by f-tests for non-parallelism, non-linearity, and significance of response in PLA 3.0 (Stegmann Systems GmbH, Raiffeisenstr, Germany).

## Results

### LMWF5A suppresses distinct pro-inflammatory cytokines in LPS-stimulated PBMC

We have previously shown that LMWF5A exhibits anti-inflammatory properties with an ≈35% inhibition of TNFα from LPS-induced human ex vivo PBMC cultures [22]. A 48-plex ELISA array of cytokines and chemokines was employed to assess broader effects in the same model. Consistent with historical findings, LMWF5A treatment resulted in a significant (p ≤ 0.05) 38 ± 6.7% inhibition or 0.62 ± 0.07-fold change in the measured OD signal for TNFα as compared to controls (Table 1). Also observed in conditioned media from LMWF5A-treated cells was significant suppression of CXCL9 (0.48 ± 0.01 fold change), CXCL10 (0.36 ± 0.15-fold change), CXCL11 (0.58 ± 0.08-fold change), IL-1β (0.71 ± 0.05-fold change), and IL-12 (0.34 ± 0.07-fold change) (Table 1). These findings demonstrate that in addition to TNFα, LMWF5A treatment of LPS-stimulated PBMC results in a distinct cytokine signature, with reduced release of specific pro-inflammatory signals after 24 h in culture.

**Table 1:** Pro-inflammatory cytokines and chemokines significantly suppressed by LMWF5A in LPS-stimulated PBMC

Cytokine/chemokine	Gene symbol	Fold change
C-X-C motif chemokine ligand 9	CXCL9	0.48 ± 0.01
C-X-C motif chemokine ligand 10	CXCL10	0.36 ± 0.15
C-X-C motif chemokine ligand 11	CXCL11	0.58 ± 0.08
Interleukin 1 beta	IL1β	0.71 ± 0.05
Interleukin 12 p40 and p70	IL12A and B	0.34 ± 0.07
Tumor necrosis factor alpha	TNFA	0.62 ± 0.07

PBMC were exposed to saline or LMWF5A and then stimulated with 100 ng/ml LPS for 24 h. Cytokine release was determined by ELISA array, and data are presented as fold change (OD ± STD, n = 3, p-value =  ≤ 0.05)

### Pathway enrichment analysis of LMWF5A differentially expressed gene sets identifies associations with CD4+ Th1 inflammatory and M1 polarization markers

Next, gene set enrichment analysis was used to gain mechanistic insight into the biologic processes underlying the identified differentially expressed cytokines by querying Enrichr using cytokine gene symbols [27, 28]. As expected for our LPS-stimulated PBMC model, the two most significant enrichment terms returned from the Wikipathways library involve LPS-related Toll-like receptor signaling pathways (Pathway#s WP75 and WP1449) (Table 2). However, these are closely followed by overrepresentation in pathway terms for allograft rejection as the result of CD4+ T cell differentiation (Pathway# WP2328), the differentiation or polarization of innate lymphoid progenitor cells (Pathway# WP3893), and the AhR signaling pathway (Pathway# WP2873) (Table 2). The top three pathways identified in the Biocarta library are associated with IL-2/IFNγ/STAT-induced Th1 differentiation (Systemic names M6231 and M4319) and the activation of NF-κB (Systemic name M2821) (Table 2). Interestingly, Biocarta also found significant overrepresentation in two well-known anti-inflammatory pathways: the PPARγ-related obesity pathway (Systemic name M22017) and the IL-10/JAK/STAT signaling pathway that result in the repression of TNFα, IL-1, and IL-6 (Systemic name M6778) (Table 2). Finally, when mined by literature in the Elsevier pathway collection, M1 macrophage polarization is associated with the LMWF5A gene set (Table 2). Together these findings suggest that the cytokine genes found to be influenced by LMWF5A treatment of LPS-stimulated PBMC are linked to classical CD4+ Th1 T cell activation, M1 macrophage polarization phenotypes, and the modulation of pro- and anti-inflammatory pathways, including PPARγ, AhR, NF-κB, and STAT.

**Table 2:** Enrichr gene enrichment analysis of the LMWF5A-reduced cytokine signature using Wikipathways, Biocarta, and Elsevier libraries

Index	Name	P-value
**Wikipathways 2019 human**		
1	Toll-like Receptor Signaling Pathway WP75	7.81E−17
2	Regulation of toll-like receptor signaling pathway WP1449	6.72E−16
3	Allograft Rejection WP2328	4.56E−14
4	Development and heterogeneity of the ILC family WP3893	1.88E−10
5	Aryl Hydrocarbon Receptor Pathway WP2873	8.53E−10
6	Cytokines and Inflammatory Response WP530	6.80E−08
7	Resistin as a regulator of inflammation WP4481	1.43E−07
8	Fibrin Complement Receptor 3 Signaling Pathway WP4136	2.03E−07
9	Type II interferon signaling (IFNG) WP619	2.03E−07
10	Lung fibrosis WP3624	1.03E−06
**Biocarta 2016**		
1	NO2-dependent IL 12 Pathway in NK cells Homo sapiens h no2il12Pathway M6231	3.78E−06
2	IL12 and Stat4 Dependent Signaling Pathway in Th1 Development Homo sapiens h IL12Pathway M4319	1.10E−05
3	NFkB activation by Nontypeable Hemophilus influenzae Homo sapiens h nthiPathway M2821	4.24E−05
4	Signal transduction through IL1R Homo sapiens h il1rPathway M12095	6.58E−05
5	Visceral Fat Deposits and the Metabolic Syndrome Homo sapiens h vobesityPathway M22017	2.80E−03
6	SODD/TNFR1 Signaling Pathway Homo sapiens h soddPathway M2699	3.15E−03
7	IL-10 Anti-inflammatory Signaling Pathway Homo sapiens h il10Pathway M6778	4.54E−03
8	Stress Induction of HSP Regulation Homo sapiens h hsp27Pathway M2587	4.89E−03
9	Cadmium induces DNA synthesis and proliferation in macrophages Homo sapiens h cdMacPathway M4388	5.59E−03
10	TNFR1 Signaling Pathway Homo sapiens h tnfr1Pathway M3618	5.94E−03
**Elsevier**		
1	Macrophage M1 Lineage	1.42E−15
2	Proteins Involved in Psoriasis	3.54E−15
3	IL6/IL12 Signaling Activates Immune System in Multiple Sclerosis	6.74E−15
4	Toll-like Receptors in beta-Cell	1.08E−12
5	CCR1 Expression Targets	9.19E−11
6	Nociception Expression Targets Signaling	3.62E−10
7	Toll-like Receptors Act through MYD88-TIRAP Signaling	5.29E−10
8	Dendritic Cells Function in Psoriasis	6.45E−10
9	Dendritic Cell Function in Ulcerative Colitis	8.53E−10
10	Hashimoto's Thyroiditis	1.31E−09

### Transcription factor enrichment analysis suggests that LMWF5A treatment involves changes in NF-kB and STAT activity

To identify transcription factors that are potentially responsible for the LMWF5A-reduced cytokine signature, differentially abundant cytokines were input into Chea3 [29]. When sorted by mean rank, the most highly associated transcription factor predicted to regulate this set of cytokines is basic leucine zipper transcription factor ATF-like 3 (BATF3), which heterodimerizes with AP-1 to function as a transcriptional repressor and may play a role in the fate of T-cell differentiation [30] (Table 3). Importantly, pro-inflammatory NF-κB family member subunits (NFKB2 and REL) and STAT1 are also represented (Table 3). In support of the initial rankings, the NF-κB subunits RELA, NFKB2, and RELB as well as STAT1 are overrepresented when assembled by gross number of overlapping genes (Table 3). In addition, sorting by overlapping genes indicates that most of the genes submitted have upstream c-Jun AP-1 promoter elements (Table 3). Thus, these data imply that the cytokines that are reduced by LMWF5A in PBMCs are, in part, regulated by NF-κB, STAT, and AP-1.

**Table 3:** Chea3 transcription factor enrichment analysis of the LMWF5A-reduced cytokine signature

Rank	Transcription factor	Mean rank	Overlapping genes
**Sorted by mean rank**			
1	Basic leucine zipper transcription factor, ATF-like 3 (BATF3)	10.33	4
2	Nuclear factor of kappa light polypeptide gene enhancer in B-cells 2 (p49/p100) (NFKB2)	17.25	5
3	Ets variant 3-like (ETV3L)	17.33	3
4	Interferon regulatory factor 8 (IRF8)	24	3
5	v-rel avian reticuloendotheliosis viral oncogene homolog (REL)	25	3
6	Zinc finger protein 267 (ZNF267)	32	3
7	Signal transducer and activator of transcription 1, 91 kDa (STAT1)	33.83	5
8	Musculin (MSC)	38	3
9	Basic leucine zipper transcription factor, ATF-like (BATF)	38.2	5
10	Early growth response 2 (EGR2)	40.33	3
**Sorted by overlapping genes**			
93	v-rel avian reticuloendotheliosis viral oncogene homolog A (RELA)	252.7	7
409	Jun proto-oncogene (JUN)	530	5
354	Estrogen receptor 1 (ESR1)	490.8	5
2	Nuclear factor of kappa light polypeptide gene enhancer in B-cells 2 (p49/p100) (NFKB2)	17.75	5
7	Signal transducer and activator of transcription 1, 91 kDa (STAT1)	34.67	5
28	Zinc finger, BED-type containing 2 (ZBED2)	143.7	5
67	v-rel avian reticuloendotheliosis viral oncogene homolog B (RELB)	215	5
95	Ets variant 7 (ETV7)	257.3	5
242	SP140 nuclear body protein (SP140)	412	5
314	Basic leucine zipper transcription factor, ATF-like 2 (BATF2)	463.3	5

### LMWF5A reduces transcription factor/DNA-binding activity

**Fig. 1:**
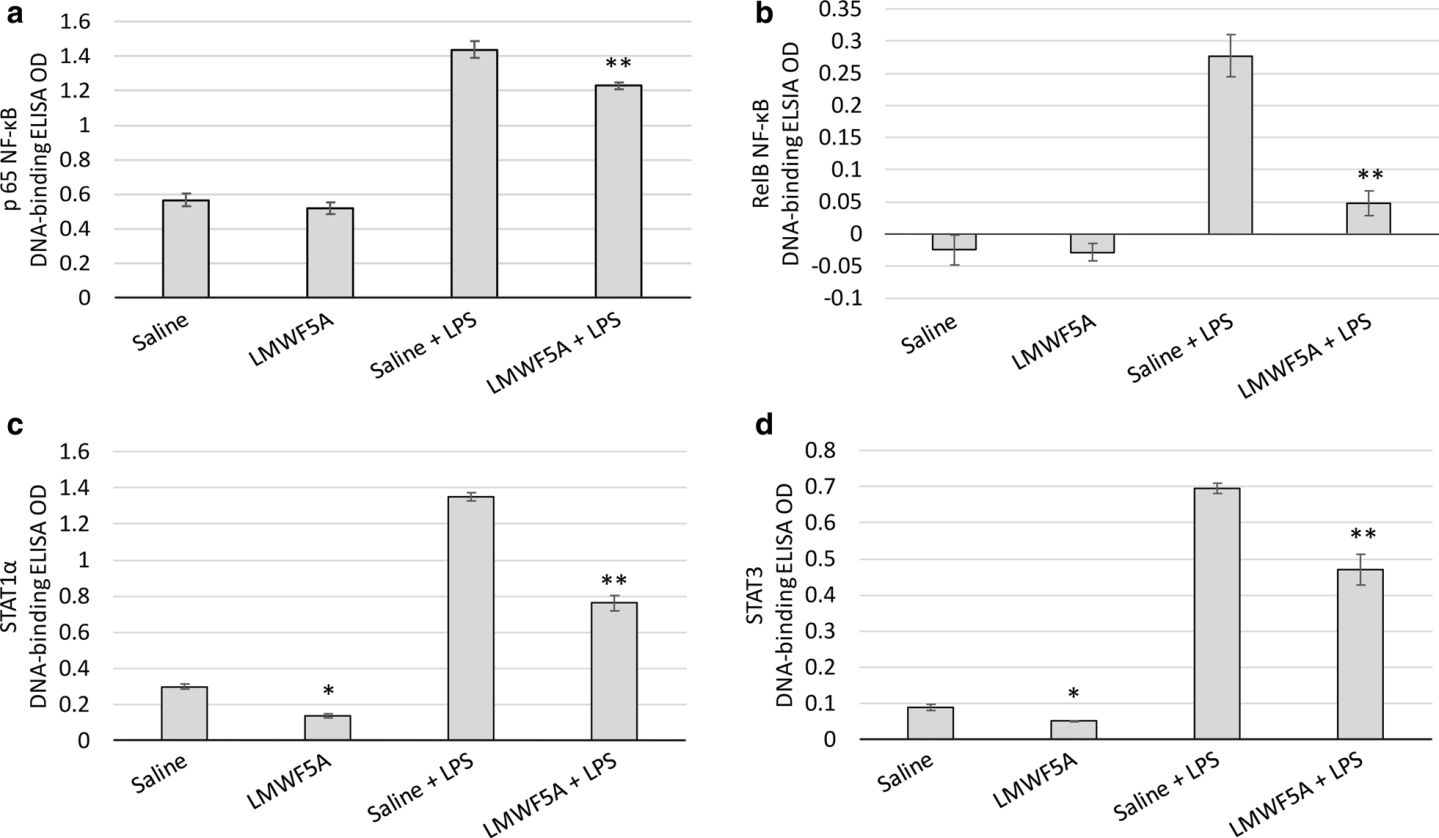
Representative transcription factor/DNA-binding ELISA results. PBMC exposed to saline or LMWF5A were stimulated with 100 ng/ml LPS or left unstimulated. After 24 h, nuclear protein was harvested and subjected to DNA-binding ELISAs for p65 NF-κB (**a**), RelB NF-κB (**b**), STAT1α (**c**), and STAT3 (**d**). Data are presented as OD ± STD (n = 3). * indicates p ≤ 0.05 versus unstimulated saline control, and ** indicates p ≤ 0.05 versus LPS-stimulated saline control

The regulatory picture provided by enrichment analysis suggests that LMWF5A treatment of LPS-stimulated PBMC results in reduced Th1/M1 differentiation or activation through attenuation in the activity of hallmark pro-inflammatory TFs, such as AP-1, NF-κB, and STAT. To test this hypothesis, nuclear protein extracts were collected from unstimulated and LPS-stimulated PBMC cultured in the presence of saline or LMWF5A for 24 h and TF activation was assessed by specific DNA-binding ELISAs. Phosphorylated c-Jun antibody was chosen for ELISA quantification to represent AP-1 activation, but no changes in optical density (OD) were observed between diluent controls and LMWF5A treatment groups (data not shown). However, as presented in Fig. 1a and b, significant reductions in ELISA OD measurements for the canonical p65 and non-canonical RelB NF-κB subunits were observed in LPS-stimulated, LMWF5A-treated nuclear protein samples. In addition, STAT1α and STAT3 DNA binding was reduced in the nuclear compartment in both unstimulated and LPS-stimulated PBMC following LMWF5A treatment (Fig. 1c and d).

**Fig. 2:**
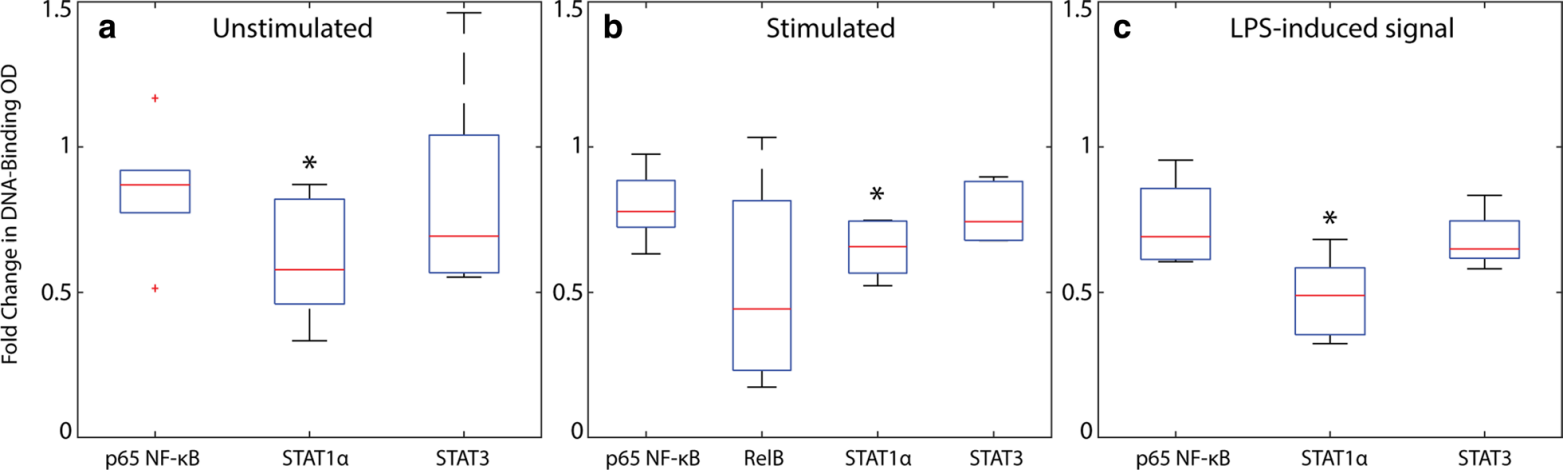
Box plots of LMWF5A-induced fold changes in nuclear transcription factor/DNA binding. Nuclear protein extracts were collected from PBMC exposed to saline or LMWF5A and stimulated with 100 ng/ml LPS or diluent control for 24 h. Transcription factor activity was then evaluated by DNA-binding ELISA, and fold changes in OD measurements were calculated for saline versus LMWF5A treatment groups for unstimulated nuclear protein samples (**a**), LPS-stimulated nuclear protein samples (**b**) and LPS-induced activation signal with unstimulated OD subtracted (**c**). Data are presented as fold change in OD ± IQR (n = 4–6). red line = median fold change, and * indicates p ≤ 0.05 versus hypothetical 1.0-fold change

To gauge the magnitude and repeatability of these responses, fold changes in OD between saline controls and LMWF5A treatments for 4–6 independent experiments were then compared to a hypothetical fold change of 1.0 for both unstimulated and LPS-stimulated cells. In addition, fold changes in the LPS-induced activation signal, adjusted to account for constituent DNA-binding activity, were also calculated by subtracting the unstimulated OD from total OD in LPS activation treatment groups. Due to the complex nature of TF DNA-binding activity and the fact that some of the investigated TFs appear to depend on activation-induced expression (i.e. RelB, Fig. 1b), both of which may impact distributions, non-parametric tests were used to test for significance. For p65, fold change in activity trends to be lower with LMWF5A treatment compared to the saline control in unstimulated cultures (Median = 0.87, Interquartile Range [IQR] = 0.79 to 0.92, p = 0.14), LPS-stimulated cultures (Median = 0.78, IQR = 0.75 to 0.85, p = 0.06), and the LPS-induced activation signal (Median = 0.69, IQR = 0.62 to 0.81, p = 0.10) (Fig. 2a–c). Interestingly, RelB activity was not detected in unstimulated cells. As a result, fold change in unstimulated treatment groups could not be calculated and the LPS-induced activation signal is reflected in the stimulated fold change for this NF-κB subunit. The biologic implications of this observation suggest that RelB must be expressed following stimulation or that this subunit is highly sequestered in the cytoplasm, which could explain the broad distribution in LMWF5A-induced fold change observed (Median = 0.44, IQR = 0.26 to 0.71, p = 0.20, Fig. 2b). As for STAT1α, LMWF5A treatment results in a significant reduction in the OD measurement quantifying DNA binding in unstimulated cells (Median = 0.58, IQR = 0.47 to 0.77, p = 0.036), stimulated cells (Median = 0.66, IQR = 0.58 to 0.73, p = 0.036), and the LPS-induced activation signal (Median = 0.49, IQR = 0.38 to 0.57, p = 0.036) (Fig. 2a–c). When STAT3 activity was evaluated, no significant change was observed in unstimulated cells (Median = 0.69, IQR = 0.57 to 0.90, p = 0.59) while stimulated cells (Median = 0.74, IQR = 0.68 to 0.88, p = 0.059) and the LPS-induced activation signal (Median = 0.65, IQR = 0.63 to 0.72, p = 0.059) trends to exhibit reduced DNA-binding in LMWF5A treatment groups (Fig. 2a–c). These findings suggest that LMWF5A reduces STAT1α activity, as measured by protein binding to specific DNA motifs, 24 h after treatment. Furthermore, LMWF5A may also reduce, to a lesser degree, the detectable DNA-binding activity of STAT3 as well as p65 and RelB NF-κB family members subunits, that may prove non-parametrically significant with more replication.

### LMWF5A reduces NF-κB luciferase reporter activity

**Fig. 3:**
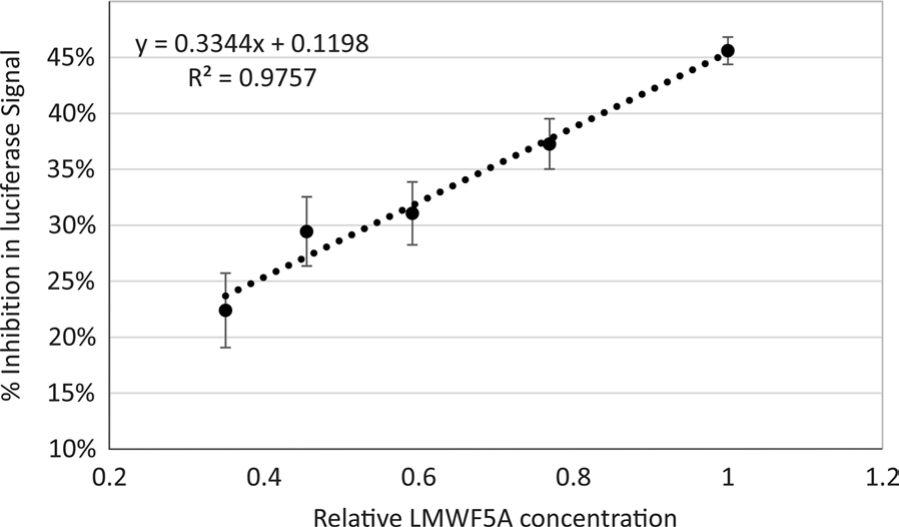
Representative LMWF5A % inhibition dose response in NF-κB reporter luciferase activity. HEK293 NF-κB luciferase reporter cells were treated with saline or serial dilutions of LMWF5A for 24 h and then stimulated with 2 ng/ml TNFα for 2 h. Luciferase expression and release into the culture medium was then determined by luminescence. Data are presented as % inhibition of luciferase activity for LMWF5A-treated, TNFα-stimulated cells versus saline-treated, TNFα-stimulated controls

NF-κB signaling is the culmination of hetero- and homodimers, consisting of five family member TFs, that can form a least 12 distinct combinations [31]. To address these complexities and capture the broader impact of LMWF5A on NF-κB signaling, a luciferase reporter system was employed. Because PBMC present technical difficulties for this model, a stably transfected NF-κB promoter-driven HEK293 reporter line (Invivogen HEK-Dual TNFα) was purchased and grown under selection conditions. The stably transfected construct consists of Lucia luciferase under the control of an IFN-β minimal promoter fused to five copies of the NF-κB consensus translational response element and three copies of the c-Rel binding site. HEK-Dual TNFα cells were cultured for 24 h with saline or serially diluted LMWF5A, and luciferase expression was evaluated 2 h after stimulation. Because HEK293 cells do not express innate pattern-recognition machinery in a similar fashion to PBMC, these cells were alternatively stimulated with 2 ng/ml TNFα, instead of LPS, to trigger robust signaling and activation. LMWF5A treatment resulted in the dose-dependent inhibition of TNFα-induced luciferase expression, ranging from 46 ± 1.2% to 21 ± 3.8%, as compared to saline-treated, TNFα-stimulated controls (R^2^ = 0.98, Fig. 3). These percent inhibition findings represent a change in the absolute luciferase luminescence units of 36,000 to 51,000 (Additional file 1: Fig. S1). When applying drug without saline dilution, LMWF5A was found to significantly inhibit reporter expression by 42 ± 6% across a total of 21 replicates (data not shown). This observation suggests that while LMWF5A does not significantly reduce p65 and RelB DNA binding, treatment effects the functional NF-κB-driven expression of luciferase.

### PPARγ and AhR antagonists reduce LMWF5A drug potency

Based on bioinformatic analysis and historical findings, we hypothesize that the biologic mechanisms surrounding LMWF5A’s anti-inflammatory activity involve activation of immunoregulatory signaling pathways and ligand-activated TFs. Our group previously reported that LMWF5A-induced AhR activation is involved in the inhibition of IL-6 release from LPS-stimulated, macrophage-like THP-1 cells [24]. In support of this, the AhR immunoregulatory pathway was identified by gene enrichment analysis in the current study using our PBMC model. Furthermore, it has been established that sodium caprylate, one of the identified active components found in LMWF5A [22], can serve as an agonist for PPARγ [32]. Importantly, a large body of evidence demonstrates that these pathways can regulate the activity of pro-inflammatory TFs as well as drive differentiation of immune cell subsets [33–35]. In addition, studies suggest that HEK293 cell lines express and are influenced by AhR and PPARγ [36, 37]. Consequently, activation of these pathways may contribute to the ability of LMWF5A to suppress the release of pro-inflammatory cytokines from activated immune cells.

Relative potency (REP) was chosen as a robust metric for examination of our hypotheses. To establish overall drug activity, a relative potency assay was developed and validated, in adherence to *USP 1032*, *1033*, and *1034* guidelines, based on the ability of LMWF5A to inhibit TNFα release in our established PBMC model. In brief, PBMC were stimulated overnight with LPS in the presence of 1.4-fold serial dilutions of LMWF5A and then TNFα release into the medium was determined by ELISA. Following this dilution scheme, LMWF5A-induced TNFα inhibition exhibits a log-linear dose response conducive to parallel-line REP calculation. Thus, separation in parallel responses can then be used to calculate the biologic activity of tested samples in relation to reference materials. Horizontal shifts in transformed TNFα inhibition dose response curves are observed when testing LMWF5A samples of known activity using this model (Additional file 2: Fig. S2A–C). Intermediate precision regression analysis, which assesses the accuracy and closeness of analytical samples, shows a correlation of 0.987 between expected and measured REP of reference LMWF5A samples manipulated into having differing activities (Additional file 2: Fig. S2D). These data demonstrate that this method provides a highly accurate and precise bioassay for determining changes in LMWF5A drug potency.

**Fig. 4:**
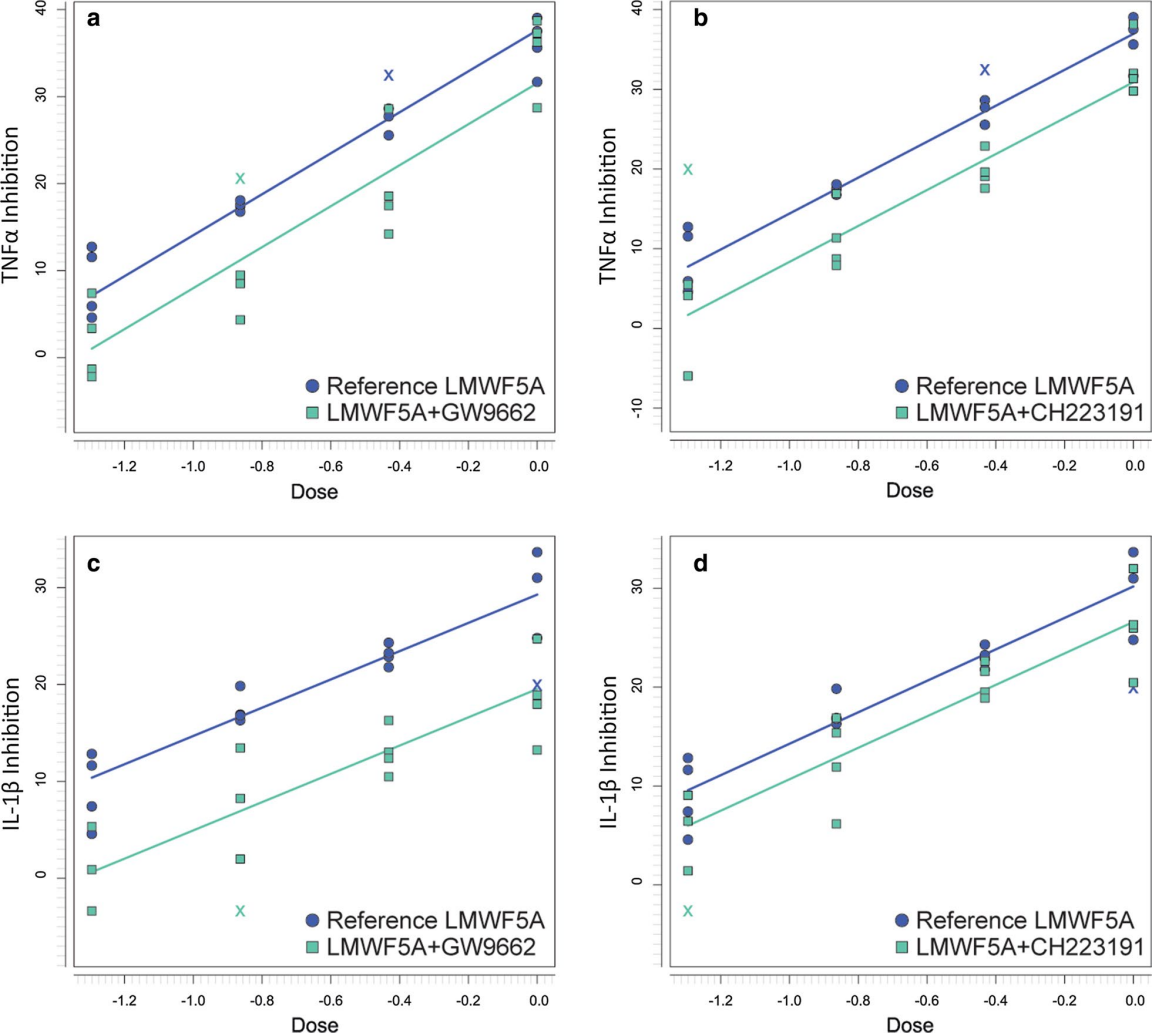
Representative TNFα and IL-1β % inhibition REP bioassays for antagonist-treated reference drug material. Dose responses in % inhibition TNFα release (**a** and **b**) or IL-1β release (**c** and **d**) for diluent control-treated reference LMWF5A and 0.5 µM GW9662 PPARγ antagonist-treated reference LMWF5A (**a** and **c**) or 0.05 µM CH223191 AhR antagonist-treated reference LMWF5A (**b** and **d**). Blue = Reference drug material dose response, Green = Antagonist-treated reference drug material dose response

The involvement of suspected pathways in the biologic activity of LMWF5A was then evaluated via this bioassay using specific antagonists for both PPARγ (GW9662, MilliporeSigma) and AhR (CH223191, MilliporeSigma). Exposure of PBMC to 0.5 µM and 0.05 µM final concentrations of GW9662 and CH223191 respectively, resulted in shifts towards reduced potency in the log-linear dose response for LMWF5A-induced TNFα inhibition (Fig. 4a and b). Additionally, IL-1β release exhibited a similar log-linear dose response in this serial dilution range, and as a result, relative potency for IL-1β was calculated. As with TNFα, PPARγ and AhR antagonism resulted in linear shifts in IL-1β dose responses that reflect a loss of drug potency (Fig. 4c and d). REP calculated for 3 independent experiments resulted in the reduction of mean REP to 0.74 ± 0.05 and 0.63 ± 0.14 for TNFα and IL-1β, respectively, when cells were treated with GW9662 in concert with LMWF5A (Table 4). Treatment with CH223191 also resulted in significant reduction in TNFα and IL-1β % inhibition potency to mean REP of 0.76 ± 0.09 and 0.72 ± 0.09, respectively. Collectively, these findings show that chemical antagonism of PPARγ and AhR transcription factors interfered with the overall anti-inflammatory activity of LMWF5A.

**Table 4:** Effect of GW9662 or CH223191 antagonism on LMWF5A drug potency

Antagonist (pathway)	Cytokine	Mean REP ± STD (95% CI)	REP 1 (95% CI)	REP 2 (95% CI)	REP 3 (95% CI)
GW9662 (PPAR)	TNFα	0.74 ± 0.05 (0.69–0.80)	0.77 (0.67–0.88)	0.69 (0.63–0.75)	0.77 (0.68–0.88)
	IL-1β	0.63 ± 0.14 (0.48–0.80)	0.51 (0.40–0.63)	0.61 (0.48–0.74)	0.79 (0.69–0.90)
CH223191 (AhR)	TNFα	0.76 ± 0.09 (0.67–0.86)	0.68 (0.57–0.78)	0.85 (0.77–0.93)	0.76 (0.67–0.86)
	IL-1β	0.72 ± 0.09 (0.62–0.82)	0.80 (0.67–0.94)	0.74 (0.58–0.82)	0.62 (0.53–0.70)

Data are presented as mean REP ± STD and 95% CI of three independent experiments

## Discussion

This investigation demonstrates that the therapeutic potential of LMWF5A involves activation of AhR and PPARγ immunoresolvent signaling pathways. Specifically, we report that specific antagonists for these TFs block the anti-inflammatory activity of LMWF5A as measured by suppression of TNFα and IL-1β release from LPS-stimulated PBMC. Here for the first time, we also present evidence that LMWF5A functions by inhibiting STAT1α DNA-binding activity as well as potentially *trans*-repressing overall NF-κB-driven expression. Hence, we propose that modulation of these pathways provides a pharmacological tool for manipulating the progression of immune responses and may contribute to the observed clinical efficacy of LMWF5A.

One of the primary findings in our study is that the AhR antagonist CH223191 attenuates LMWF5A drug potency as measured by the inhibition of both TNFα and IL-1β release. This is in agreement with a previous study in which CH223191 partially blocks LMWF5A-induced inhibition of IL-6 from macrophage-like LPS-stimulated, PMA-differentiated THP-1 cells [24]. To put this in context, the most abundant molecule found in LMWF5A is N-acetyl-tryptophan (NAT). This synthetic amino acid is added to pharmaceutical 5% human serum albumin, the starting material for LMWF5A, at high concentration (4 mM) to help stabilize the protein, with the added benefit of serving as a reactive oxygen species scavenger [38]. Subsequent oxidative breakdown of NAT results in the production of a variety of tryptophan-like metabolites, which have been identified in both HSA solutions as well as LMWF5A [24, 38]. It is also well established that enzymatic catabolism of tryptophan by indoleamine 2,3-dioxygenase (IDO) results in production of biologically active metabolites that act via AhR [11]. These data lead us to believe that NAT metabolism contributes to the biologically relevant activation of AhR by LMWF5A.

AhR is a ligand-activated member of the basic helix-loop-helix TF family. The latent form of AhR resides in the cytosol, complexed together with two heat shock protein 90 s, XAP-molecule 2, the co-chaperone p23, and potentially Src tyrosine kinase, which protects AhR from proteasomal degradation [11]. Ligand docking results in translocation of the complex to the nucleus, where AhR heterodimerizes with Ah receptor nuclear translocator (ARNT) [39]. Together, the AhR/ARNT complex provides a functional DNA-binding domain for a specific motif known as the dioxin or xenobiotic-response element (XRE) that influences the transcription of a diverse set of genes, including the xenobiotic metabolizing enzyme cytochrome P450 family-1 subfamily-A polypeptide-1 [40]. Interestingly, preliminary RNA-sequencing experiments, performed on a variety of cell types, identify cytochrome p450 genes as some of the most highly LMWF5A-regulated genes, suggesting that this pathway is indeed activated by treatment (unpublished findings).

It is now recognized that in addition to its classical transcriptional activity, AhR can repress and/or alter NF-κB activity through the formation of unique complexes that act as cofactors or cis-acting elements. NF-κB is a family of five structurally similar members, including p65 or RelA, p50, p52, c-Rel, and RelB, which can target gene expression following inflammatory activation in various homo- and hetero- dimer combinations [7]. In some studies, AhR signaling skews the combinatorial makeup of NF-κB dimers that enter the nucleus and physically bind to cognate DNA sequences. For instance, Puga et al*.* demonstrated that AhR ligand treatment of mouse cell lines results in enhanced formation of repressive p50/p50 NF-κB homodimers, which may result in a competitive reduction in inflammatory p50/RelA NF-κB heterodimer formation and binding [41]. However, this response appears to be cell type- and ligand-dependent. For example, in dendritic cells it has been observed that AhR activation inhibits overall RelA DNA binding with no apparent change in the complexing of p50, suggesting that AhR-driven NF-κB suppression can also result from of sequestering of specific subunits in the cytosol [42]. Interestingly, the possibility also exists that concomitant activation of NF-κB and AhR results in the generation of alternate immunoregulatory pathways. Several lines of evidence suggest that complexes containing AhR and RelB or RelA can serve as functional dimers that mediate the expression of pro-inflammatory cytokines or inhibit gene expression driven by XREs [43, 44]. Finally, AhR can also impact the activity of NF-κB through direct trans-repression in the presence of STAT. This was exemplified in a study showing that ligand activated AhR complexes with STAT1 to physically suppresses NF-κB activity in LPS-activated peritoneal macrophages [45]. While these interactions still need to be fully elucidated, AhR activation appears to provide multiple avenues of transcriptional modulation that may explain the ability of LMWF5A to inhibit NF-κB activity and sequester STAT1α.

Extensive research chronicles the ability of AhR signaling to play a pivotal role in immunoregulation. Of note, most immune cells express AhR, which appears to increase in expression both during differentiation and upon exposure to pro-inflammatory conditions, and many inflammatory response-related genes contain upstream XREs [46]. Studies designed to evaluate the functional implications of AhR signaling on adaptive immunity reveal that activation promotes development of immunosuppressive Treg phenotypes [46]. However, AhR also conveys activity in innate immune responses. For example, LPS-induced production of IL-6, TNFα, and IL-12 is elevated from AhR^−/−^ peritoneal macrophages as compared to wild-type controls [47]. Animal models also demonstrate the importance of AhR to the overall immune response. AhR-deficient mice exhibit lower IL-10 levels and augmented levels of IL-12 and IFNγ following T-cell receptor activation or microbial challenge [46]. Moreover, the tryptophan-metabolizing enzyme IDO1 and the tryptophan metabolite kynurenine, together with the anti-inflammatory cytokine TGFβ, are required for the development of endotoxin tolerance in murine models [48]. Taken together, these studies have established that AhR signaling is a fundamental part of immune suppression and end-stage resolution, providing an interesting therapeutic target for immunomodulation.

Another pivotal observation presented in this report is that PPARγ antagonism with GW9662 also partially blocks LMWF5A-induced cytokine effects. As previously noted, indirect relationships between LMWF5A and PPARγ signaling have been established in previous investigations. The first indication that this pathway is activated by LMWF5A was provided when sodium caprylate was identified an active component [22] because medium chain fatty acids, including caprylate, can serve as a PPARγ agonist [32]. Moreover, subsequent studies have shown that release of known endogenous lipid mediator ligands of PPARγ, such as PGD2 and 15d-PGJ2, are potentiated by LMWF5A in PBMC [23]. Activation of this pathway by LMWF5A was further substantiated in a published study demonstrating that treatment of bone marrow-derived mesenchymal stem cells results in approximately four-fold increases in the DNA-binding activity of PPARγ and its binding partner, retinoid X receptor as measured by hybridization in nuclear extracts [25]. Together, these data suggest that PPARγ signaling is involved in the biologic activity of LMWF5A; however, here for the first time, we provide direct evidence that this pathway is indeed crucial to the biologic activity of LMWF5A.

PPARs comprise a family of nuclear hormone receptors that are structurally similar to steroid receptors and regulate transcription through a diverse set of mechanisms [14, 34, 49]. In their classical mechanism, this family responds to the presence or absence of signal or ligand by inducing or repressing, respectively, transcription of a multitude of gene targets associated with fatty acid oxidation and metabolism [14, 34, 49]. In the absence of ligand, PPAR resides in the nucleus complexed with co-repressors to repress the expression of genes via a mechanism termed ligand-independent *trans*-repression [14, 34, 49]. Ligand binding—in the case of LMWF5A, possibly caprylate or 15d-PGJ2 recognition—leads to ligand-dependent *trans*-activation, which commences with conformational changes that cause disassociation from co-repressors and allow for heterodimerization to its PPAR response element binding partner, the retinoid X receptor [14]. In addition, PPARs can evoke anti-inflammatory outcomes through ligand-dependent *trans*-repression in several distinct fashions. For example, PPARs have been demonstrated to directly interact with the p65 and p50 subunits of NF-κB as well as the c-Jun subunit of AP-1, preventing their ability to bind to their DNA response elements [50, 51]. Moreover, PPAR can also reduce NF-κB and AP-1 activity by modulating expression and activity of key upstream proteins and enzymes, such as inhibitor of nuclear factor kappa B, c-Jun N-terminal kinase, and p38 mitogen-activated protein kinase [14]. Studies of PPAR agonists have also demonstrated the functional outcome of these activities, as they antagonize AP-1, STAT, and NF-κB in LPS-stimulated macrophages [52]. Thus, via *trans*-repression, PPAR activation may suppress pro-inflammatory TF activity and production of key inflammatory cytokines, including TNFα, IL-12, and IL-1β.

PPARs are widely known as drug targets for diabetes due to their inherent regulation of genes related to glucose metabolism and fatty acid storage, but have more recently become prominent for their anti-inflammatory activity [49]. Several studies have shown that the PPARγ agonists, such 15d-PGJ2, ciglitazone, and pioglitazone, confer protection in neuroinflammatory and sepsis animal models through the inhibition of STAT, AP-1, and NF-κB activity as well as reduced Th1 differentiation [53–55]. Furthermore, one of the hallmarks of the transition into immune resolution is the class-switching of eicosanoids to PGD2 and 15d-PGJ2 isoforms [49]. In support of this aspect of PPARγ biology, agonists appear to attenuate inflammatory pain responses and promote tissue repair by driving the conversion of macrophages to the M2 phenotype [56, 57]. It has also been well documented that PPARγ ligands negatively regulate the production of pro-inflammatory cytokines, such as TNFα, IL-1β, IL-2, IFNγ, and CXCL10, from a diverse set of immune cells, including macrophages, dendritic cells, and T cells in cell culture [14]. Conversely, the Th2 cytokine IL-4 appears to mediate its anti-inflammatory activities by upregulating PPARγ expression, and full-blown inflammation requires down-regulation of PPARγ [58, 59]. Therefore, PPARγ is another intriguing target for treating inflammatory conditions.

There are several limitations of this investigation that should be noted. An important caveat for the interpretation of this study is the use of a single donor of PBMC cells exhibiting strong drug response. Previously we have demonstrated that in a similar model, LMWF5A exhibits the ability to inhibit LPS-induced TNFα release from a diverse set of PBMC donor lots by 26–46% [22]. While our prior data suggest that LMWF5A affects a broad range of genotypes, future work should explore if the findings presented in this report influence the donor-to-donor variance observed in these models. In addition, the cytokine array utilized resulted in the identification of a limited number of inflammatory-associated protein markers for enrichment analysis. While this may result in a potentially biased set of genes, already linked to pro-inflammatory TFs, the resulting hypotheses generated were subject to subsequent confirmation by molecular means.

## Conclusion

**Fig. 5:**
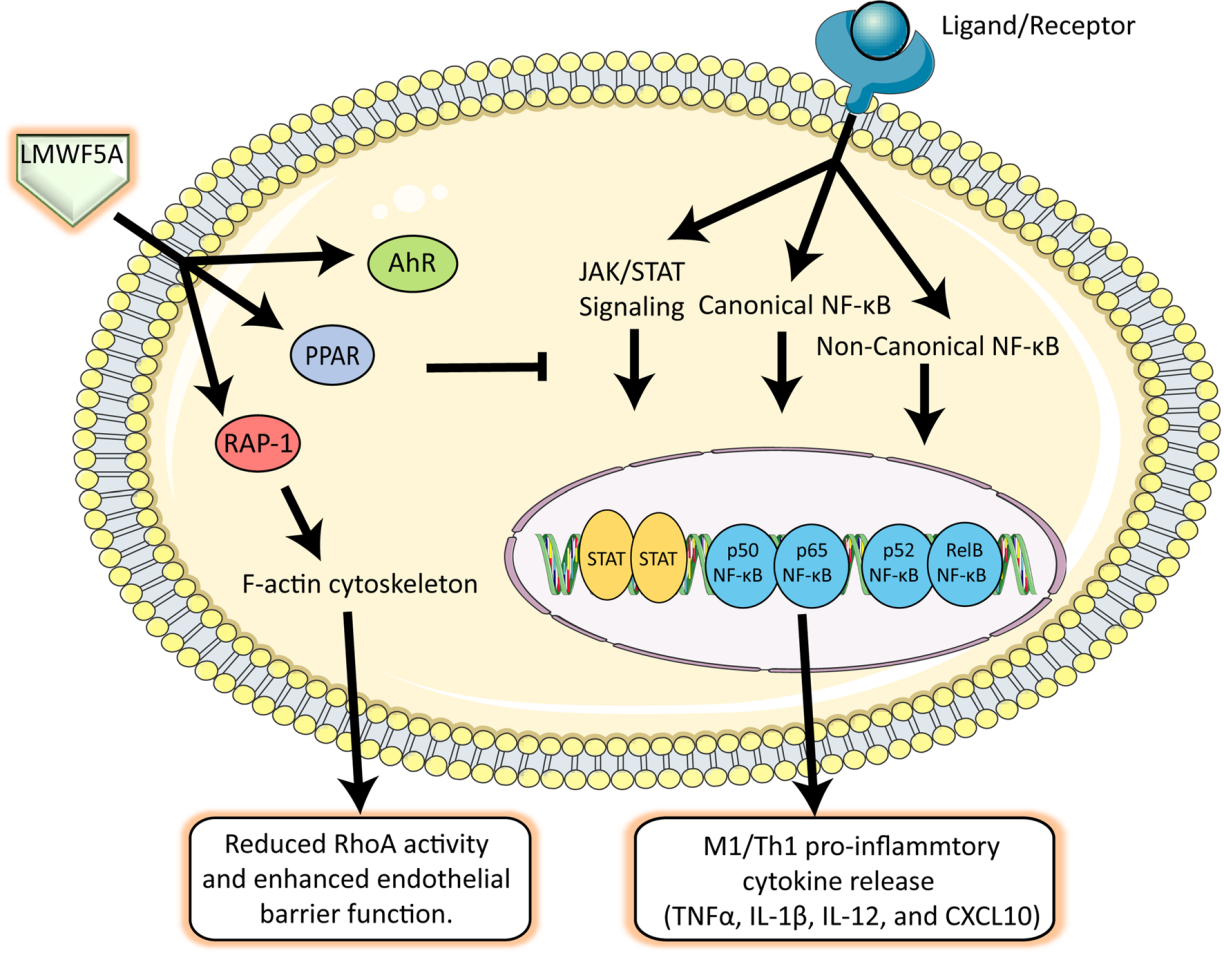
LMWF5A mechanisms of action. The activation of JAK/STAT as well as both canonical and non-canonical NF-κB signaling pathways leads to the secretion of inflammatory M1 and Th1 cytokines associated with inflammatory disease and tissue damage. Treatment with LMWF5A results in the activation of PPARγ and AhR, which may repress STAT and NF-κB transcriptional activity. In addition, LMWF5A activates RAP-1 small GTPase, which aids in reducing RhoA GTPase activity and promotes f-actin cytoskeletal rearrangements that enhance endothelial barrier function. Together, these mechanisms of actions may reduce leukocyte extravasation and impede M1 polarization to restore hemostasis in chronic settings

In conclusion, this study expands our knowledge on the mechanisms of action of LMWF5A. We have previously documented that LMWF5A can modulate both the activity of small GTPases, such as RAP-1, Rac-1, and RhoA, as well as alter cytoskeletal post-translational modifications and cellular arrangements [25, 60, 61]. These activities are attributed to the ability of LMWF5A reduce both adaptive immune cytokine release and endothelial permeability [60, 61]. The findings presented in the current study provide new evidence on the mechanisms of action of LMWF5A, specifically that AhR and PPARγ signaling pathways are directly involved in LMWF5A-induced suppression of cytokine release in LPS-stimulated PBMC. Importantly, the distinct shift in M1/Th2 pro-inflammatory cytokine programming seen following LPS stimulation in this model suggests that LMWF5A is not simply acting as an anti-inflammatory, but in a polypharmacologic manner by exerting pro-resolving activities. Together, the known mechanisms of actions of LMWF5A may reduce leukocyte extravasation and impede M1 polarization to restore hemostasis in dysregulated inflammatory settings (Fig. 5). Exploiting these pathways and mechanisms with LMWF5A may hold promise not only in the treatment of osteoarthritis, but also in the treatment of a variety of inflammatory conditions as far ranging as the cytokine release syndromes seen in respiratory infections, inflammatory bowel disease, and central nervous system inflammation. These data provide previously undescribed mechanistic insight into the biologic activities of LMWF5A and should drive the informed repositioning of this novel biologic drug for the treatment of other inflammatory conditions.

## Supplementary information

**Additional file 1: Fig. 1.** Representative LMWF5A dose response in NF-κB reporter luciferase activity. HEK293 NF-κB luciferase reporter cells were treated with serial dilutions of LMWF5A for 24 h and then stimulated with 2 ng/ml TNFα for 2 h. Luciferase expression and release into the culture medium was then determined by luminescence. Data are presented as luciferase luminescence units.

**Additional file 2: Fig. 2.** Intermediate precision of TNFα REP bioassay. A-C) Representative transformed % inhibition LPS-induced TNFα release versus nominal drug concentration for reference drug material (blue) versus test material (green) for 50% (0.50) drug sample (A), 100% (1.00) drug sample (B), and 200% (2.00) drug sample (C). D) Regression analysis of measured REP versus theoretical REP intermediate precision (n = 5 for 0.5; n = 6 for 0.75, 1.00, 1.50, and 2.00).

## Data Availability

The data analyzed during in this report are included in published articles or available from the corresponding author on reasonable request.

## Abbreviations

LMWF5A: Low molecular weight fraction of human serum albumin
M1: Classically activated macrophage
M2: Alternatively activated macrophage
Th1: CD4+ T helper 1 T cell
Th17: CD4+ T helper 17 T cell
Th2: CD4+ T helper 2 T cell
TF: Transcription factor
NF-κB: Nuclear factor kappa-light-chain-enhancer of activated B cells
p50: NF-κB subunit p50
RelA: NF-κB subunit p65
RelB: NF-κB subunit RelB
p52: NF-κB subunit p52
c-Rel: NF-κB subunit c-Rel
NFKB2: NF-κB subunit p100
STAT: Signal transducer and activator of transcription
AhR: Aryl hydrocarbon receptor
PPAR: Peroxisome proliferator-activated receptor
AP-1: Activator protein
LPS: Lipopolysaccharide
PBMC: Peripheral blood mononuclear cells
PMA: Phorbol 12-myristate 13-acetate
TNFα: Tumor necrosis factor alpha
IFNγ: Interferon gamma
IL-1β: Interleukin 1 beta
IL-2: Interleukin 2
IL-6: Interleukin 6
IL-10: Interleukin 10
IL-12: Interleukin 12
CXCL9: C-X-C motif chemokine ligand 9
CXCL10: C-X-C motif chemokine ligand 10
CXCL11: C-X-C motif chemokine ligand 11
OD: Optical density
IQR: Interquartile Range
JAK: Janus tyrosine kinase
BATF3: Basic leucine zipper transcription factor ATF-like 3
c-Jun: AP-1 protein c-Jun gene
REP: Relative potency
NAT: N-acetyl-tryptophan
IDO: Indoleamine 2,3-dioxygenase
ARNT: Ah receptor nuclear translocator
XRE: Xenobiotic-response element
TGFβ: Transforming growth factor beta
COX-2: Prostaglandin-endoperoxide synthase 2
PGE2: Prostaglandin E_2_
PGD2: Prostaglandin D_2_
15d-PGJ2: 15-Deoxy-Δ-12,14-Prostaglandin J_2_
GTPase: Guanosine triphosphate hydrolase
RAP-1: Ras-related protein RAP-1
RhoA: Ras homolog family member A
Rac1: Rac family small GTPase 1
