# Supplementary figures and images for: LMWF5A suppresses cytokine release by modulating select inflammatory transcription factor activity in stimulated PBMC
Source: J Transl Med. 2020 Nov 30;18:452. doi: 10.1186/s12967-020-02626-z (PMC7702209; doi:10.1186/s12967-020-02626-z)

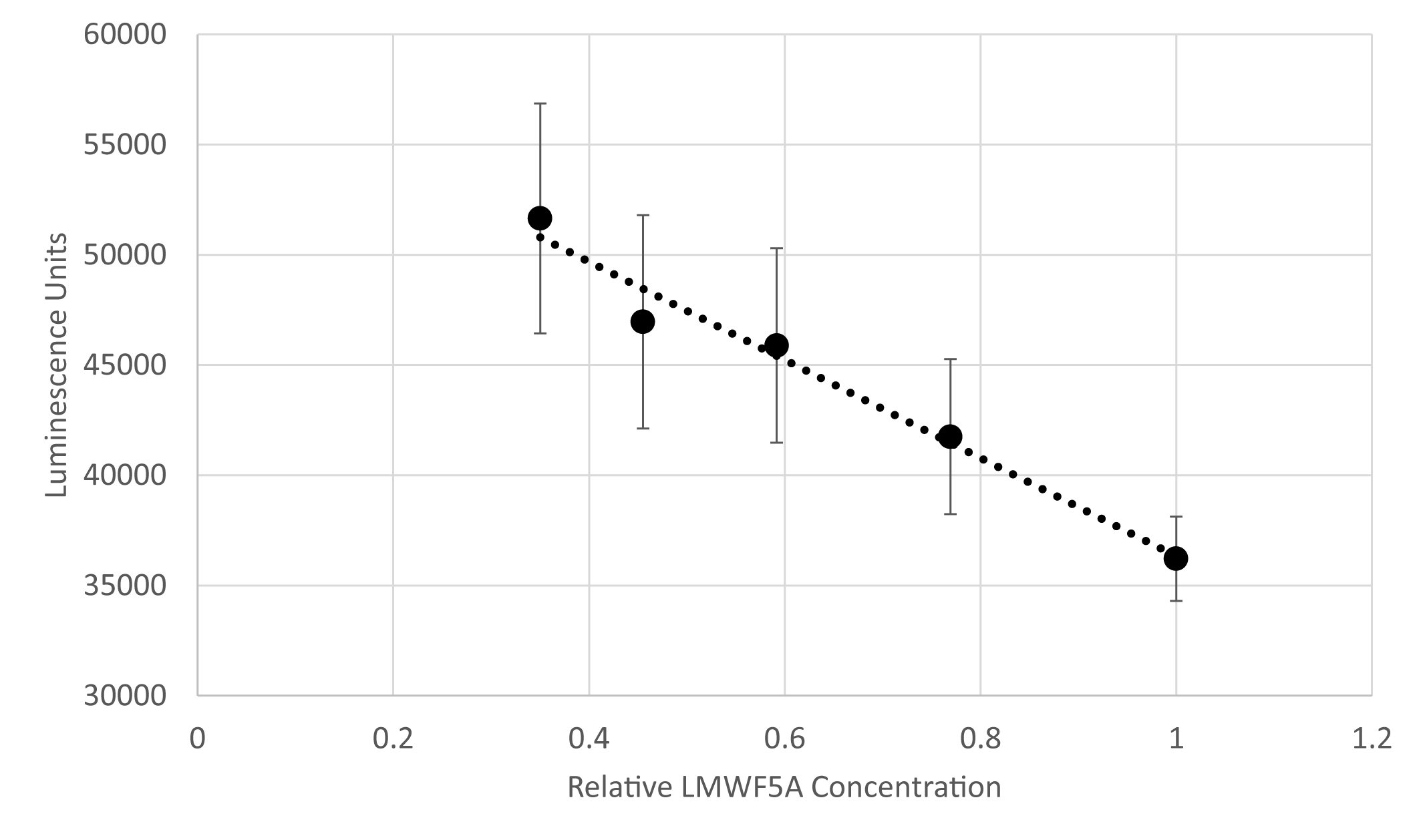

Supplement: Supplementary file 1 — Additional file 1: Fig. 1. Representative LMWF5A dose response in NF-κB reporter luciferase activity. HEK293 NF-κB luciferase reporter cells were treated with serial dilutions of LMWF5A for 24 h and then stimulated with 2 ng/ml TNFα for 2 h. Luciferase expression and release into the culture medium was then determined by luminescence. Data are presented as luciferase luminescence units. [file 12967_2020_2626_MOESM1_ESM.tif]

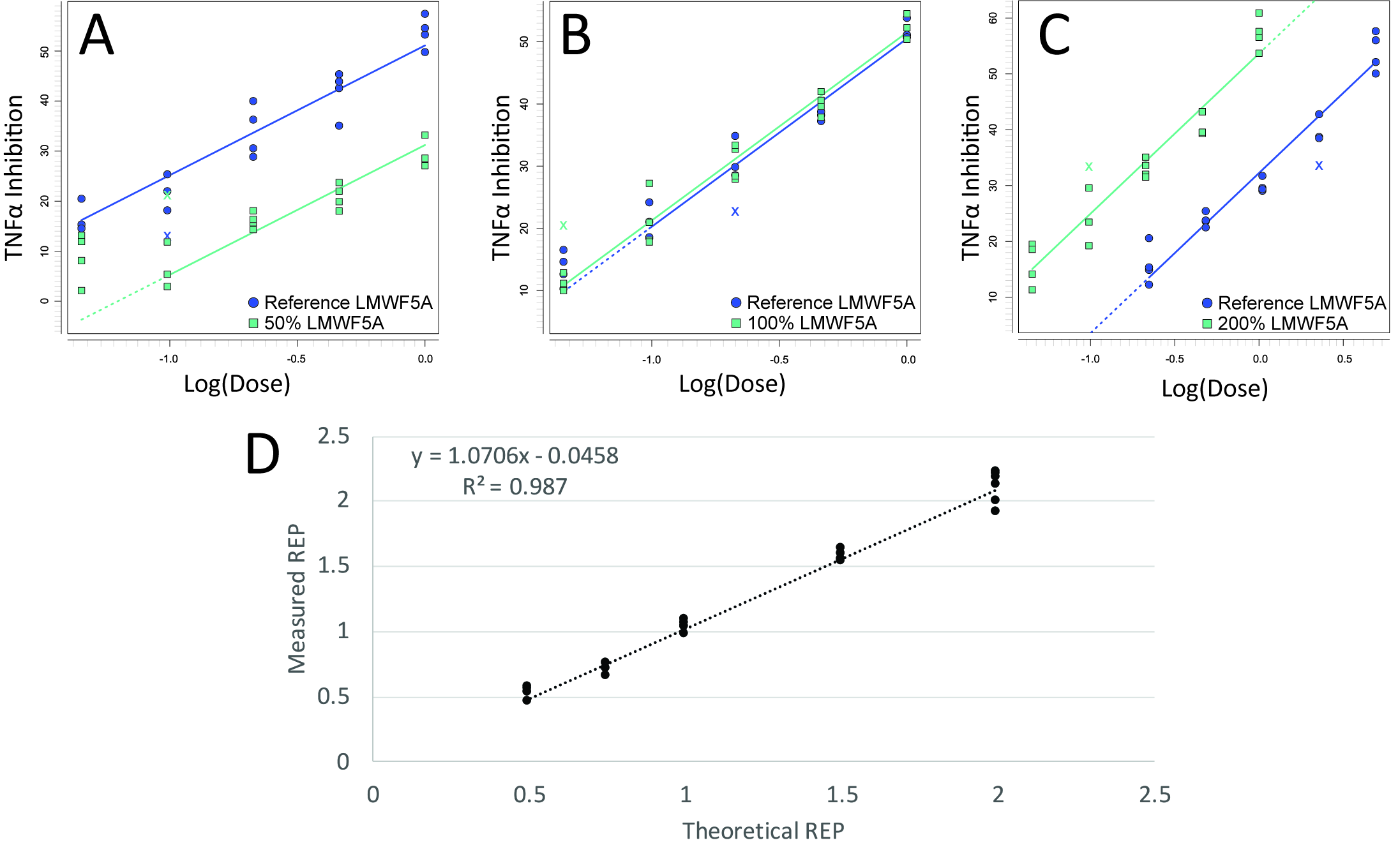

Supplement: Supplementary file 2 — Additional file 2: Fig. 2. Intermediate precision of TNFα REP bioassay. A-C) Representative transformed % inhibition LPS-induced TNFα release versus nominal drug concentration for reference drug material (blue) versus test material (green) for 50% (0.50) drug sample (A), 100% (1.00) drug sample (B), and 200% (2.00) drug sample (C). D) Regression analysis of measured REP versus theoretical REP intermediate precision (n = 5 for 0.5; n = 6 for 0.75, 1.00, 1.50, and 2.00). [file 12967_2020_2626_MOESM2_ESM.tif]
